# Complete Genome Sequences, before and after Mammalian Cell Culture, of Zika Virus Isolated from the Serum of a Symptomatic Male Patient from Oaxaca, Mexico

**DOI:** 10.1128/genomeA.00072-17

**Published:** 2017-03-23

**Authors:** Celia Boukadida, Jesús M. Torres-Flores, Martha Yocupicio-Monroy, Elvira Piten-Isidro, Amaranta Y. Rivero-Arrieta, Yara A. Luna-Villalobos, Liliane Martínez-Vargas, Sofía L. Alcaraz-Estrada, Klintsy J. Torres, Rosalia Lira, Gustavo Reyes-Terán, Edgar E. Sevilla-Reyes

**Affiliations:** a Departamento de Investigación en Enfermedades Infecciosas, Instituto Nacional de Enfermedades Respiratorias Ismael Cosío Villegas (INER), Ciudad de México, Mexico; b Laboratorio de Virología, Escuela Nacional de Ciencias Biológicas, Instituto Politécnico Nacional, Ciudad de México, Mexico; c Posgrado en Ciencias Genómicas, Universidad Autónoma de la Ciudad de México, Ciudad de México, Mexico; d Centro Médico Nacional 20 de Noviembre, Instituto de Seguridad y Servicios Sociales de los Trabajadores del Estado, Ciudad de México, Mexico; e Unidad de Investigación Médica en Enfermedades Infecciosas y Parasitarias, UMAE Hospital de Pediatría, Instituto Mexicano del Seguro Social (IMSS), Ciudad de México, Mexico

## Abstract

Zika virus (ZIKV) is an emerging arthropod-borne flavivirus associated with severe congenital malformations and neurological complications. Although the ZIKV genome is well characterized, there is limited information regarding changes after cell isolation and culture adaptation. We isolated, and passaged in Vero cells, ZIKV from the serum of a symptomatic male patient and compared the viral genomes before and after culture. Single nucleotide polymorphisms were characteristic among serum-circulating genomes, while such diversity decreased after cell culture.

In March 2016, three days after the onset of Zika virus (ZIKV) infection symptoms, an 80-year-old male patient from Santa Maria Mixtequilla, a rural town located in Southern Oaxaca, Mexico, agreed to participate in this study by signing the informed consent forms and by donating a blood sample (protocol C16-16 approved and registered at INER). The blood serum (CIENI551_serum) was positive for ZIKV by reverse transcriptase real-time PCR (1) and another aliquot was passaged four times in Vero cells (CIENI551P4 isolate). Viral nucleic acids were enriched by filtrating 500 μL of serum or culture supernatant through a 0.45-μm filter (Millipore) followed by Turbo DNase (Ambion) and RNase I (Invitrogen) treatment. Viral RNA was extracted using the QIAamp viral RNA minikit (Qiagen) and reverse-transcribed with Superscript III reverse transcriptase (Invitrogen), followed by second-strand synthesis with the Klenow fragment (New England Biolabs) for sequence-independent amplification, as described in reference 2. Libraries were constructed with the Nextera XT DNA library preparation kit (Illumina) and sequenced using the NextSeq Illumina platform (2 × 150 bp). Human genome reads were removed from the serum-derived library by mapping against the GRCh38 reference in BWA-MEM (3), and MIRA4 was used for *de novo* assemblies (4) in a local Galaxy server implementation (5). After PCR clean-up in MarkDuplicates (http://broadinstitute.github.io/picard) and filtering for high-quality mapping reads, we reached a ZIKV CIENI551 assembly with an average read depth of 320× and a length of 10,633 bp, while the passaged isolate CIENI551P4 assembly had an average read depth of 6,800× and a length of 10,744 bp, due to better coverage on the 3′ end of the genome. The polymorphism threshold was set at 15% of all mapped high-quality reads per locus, distributed on both strands with 50 reads as the minimum depth.

The ZIKV CIENI551 serum genome contained nine polymorphic positions, six of which were nonsynonymous single nucleotide polymorphisms, and the most abundant variants corresponded to sequences reported in GenBank. In contrast, no polymorphic positions were detected in CIENI551P4.

Thus, we compared ZIKV genomes before and after mammalian cell culture. Single nucleotide polymorphisms were characteristic among the serum-circulating genomes, while such diversity decreased after cell culture, presumably reflecting the selection of the most abundant variant during cell culture.

## Accession number(s).

The consensus sequences of the ZIKV genome in serum (MEX_CIENI551_serum) and after four passages in Vero cells (MEX_CIENI551P4) have been deposited in GenBank under the accession numbers KY120349 and KY120348, respectively.
